# Combined effect of lightning impulse voltage and temperature stress on the propagation of creeping discharge of oil-impregnated paper

**DOI:** 10.1038/s41598-024-56700-3

**Published:** 2024-03-12

**Authors:** Jean Lambert Jiosseu, Stanley Vianney Foumi Nkwengwa, Ghislain Mengata Mengounou, Emeric Tchamdjio Nkouetcha, Adolphe Moukengue Imano

**Affiliations:** 1 Pure Physique Laboratory UFD MIP, University of Douala, Douala, Cameroon; 2 Research Laboratory of Industrial Computer Science and Automation Engineering, Advanced Teachers Training College for Technical Education of the University of Douala, Douala, Cameroun; 3 Laboratory of Technology and Applied Sciences, University of Douala, Douala, Cameroon

**Keywords:** Creeping discharges, Castor oil, Mineral oil, Palm kernel oil, Impregnated pressboard, Energy science and technology, Engineering

## Abstract

This article presents the results of an experiment designed to study the impact of temperature on the characteristic parameters of creeping discharges. The insulating interfaces consist of a thermally enhanced cellulose surface immersed in mineral oil, palm kernel oil methyl ester (PKOME) and castor oil methyl ester (COME). The study was carried out under a standard negative lightning impulse voltage (1.2/50 μs). The article also presents the complete algorithms for calculating the maximum extension of the discharges, the ionisation rate and the charge produced by them. The results of the study show that temperature favors the propagation of discharges and the ionisation rate. It was observed that liquids with a higher dielectric constant and high electrical conductivity were more exposed to the impact of temperature. The results show ionisation increments of 0.973%/°C, 1.093%/°C and 1.076%/°C in mineral oil (MO), COME and PKOME respectively. The maximum extension of the discharges shows a linear evolution with the applied voltage and temperature but a non-linear increment with the temperature. As for the charge produced, it shows a constant increment with temperature and voltage in each liquid. These values are (5.839%/°C, 1.977%/kV), (6.047%/°C, 2.082%/kV) and (6.177%/°C, 2.113%/kV) respectively in MO, COME and PKOME.

## Introduction

Some of the main functions of dielectric fluids in high voltage equipment are to ensure heat transfer and to reinforce electrical insulation by impregnating cellulose. For a large proportion of power transformers, for example, the insulation is of the composite type (oil/cellulose). However, although this type of insulation increases the transformer's performance, the interface between the oil and the cellulose is one of the equipment's biggest weaknesses^1^. In fact, this region is ideal for the propagation of creeping discharges. This makes it one of the biggest causes of power transformer failure in the world^2,3^. There are several reasons for this phenomenon. In general, under the effect of significant electrical stress due to voltage surges, the mismatch between the permittivity of the liquid and that of the cellulose is likely to deform the field lines, making them even more divergent and intense. This process creates leakage discharges at the liquid/cellulose interface. A long-term effect of this type of failure can create erosion and in the long term irreversible destruction on the surface or inside the cellulose.

A number of research projects have already been carried out with the aim of understanding the process giving rise to the phenomenon of creeping discharges at the liquid/solid interface and their impact factor. In general, the literature on the experimental characterisation of the phenomena of generation and propagation of creeping discharges has focused on parameters such as the maximum extension of the branches, the discharge inception voltage, the radial occupation density, the fractal parameter and many others. In most cases, the investigations carried out on this subject are directed towards the search for a substitute for mineral oils, which have been the most widely used in power transformers for decades. We can cite the work of Fri Murdiya et al.^4^ on the analysis of the behaviour of creeping discharges propagating on cellulose immersed in palm kernel and rapeseed vegetable oils in comparison with mineral oil. Their experiment shows that rapeseed oil and mineral oil produce a higher rate of ionisation than palm kernel oil. On the other hand, they show that the maximum extension of discharges is much higher in rapeseed oil and mineral oil than in palm kernel oil. This result suggests that, subject to further investigation, vegetable oils can be used as a substitute for mineral oils. In a similar experiment using rapeseed oil, soybean oil and mineral oil, Beroual et al.^5^ studied the effect of the thickness of the cellulose insulation on the maximum extension of the discharges propagating in these different liquids under DC, AC and lightning impulse voltage. Their experiment showed that there is a linear relationship between the maximum extension of the discharge and the amplitude of the applied voltage. They also showed that the effect of the discharges is greatly reduced when the thickness of the insulation is greater. They also show that the characteristics of these discharges are almost identical in the mineral oil and vegetable oils investigated. As in the previous case, this conclusion makes it possible to think about the use of these vegetable oils in power transformers as a substitute for mineral oils. Under similar experimental conditions, Sitorus et al.^6^ carried out a comparative study of the propagation of creeping discharges on cellulose insulation immersed in Jatropha vegetable oil compared with mineral oil. The conclusions drawn from their investigation are identical to those of Beroual et al.^5^, according to which the propagation of discharges is almost identical in vegetable oils and mineral oils and is strongly linked to the thickness of the solid insulator and the amplitude of the applied voltage. Other liquids such as extra virgin olive oil, olive oil, tetra-ester, rapeseed oil and mineral oil were also investigated by Reffas et al.^7^. The conclusions of their work are identical to the previous ones in terms of the propagation of discharges. They also show that the morphology and radial occupation of the discharges are closely linked to the polarity of the applied voltage and to the form of the voltage (DC, AC or LI voltage). They conclude that positive voltages are the ones that produce more risk due to the longer branch lengths than those produced by negative polarity. Xudong Li et al.^8^ investigated the effect of combined voltage stress on the impregnated cellulosic surface. Their technique consists of subjecting the interface to a combined AC and DC voltage stress and observing the behavior of the creeping discharge at different voltage levels until dielectric breakdown. Their experience shows that the distribution of discharges is strongly influenced by the DC component of the signal. They also note that even under conditions of high humidity of the impregnated cellulose, it is very difficult to create a fault on it with a system of combined AC + DC voltages. Dang et al.^9^ have also analysed the behavior of creeping discharges on a cellulose surface with stresses of this type. The difference with the previous case is that the stresses are taken independently. They also looked at the response of vegetable oils to these stresses compared with mineral oils. The conclusion of their experiment is similar to that of previous work in terms of the effect of the thickness of the cellulose insulation on the maximum extension of the discharges and the similarity of the results obtained in the mineral oil and vegetable oils investigated. However, there is a slight contradiction with the work of Xudong Li et al.^8^ on the impact of the DC component of the signal. In this case, the impact of the discharge is greater in AC. Kebbabi et al.^10^ studied the impact of the nature of the solid insulator on the propagation of creeping discharges. These include glass, phenolic resin and polycarbonate. Their analysis technique is based on determining the fractal dimension of the discharges obtained as a function of the type of insulator and their thickness. Based on their experience, they show that there is a close relationship between the fractal dimension of the discharges produced, the nature of the material and the thickness of the material. Their results establish a direct link between the physico-chemical characteristics of the materials used for insulation and the propagation of creeping discharges. This result was confirmed by similar work carried out with solid insulators such as simple glass, hardened glass and porcelain^11,12^. The main aim of this work was to establish the relationship between the type of insulation system and the propagation of creeping discharges. On the other hand, the aim was to study new prototypes of plant-based insulating materials that could be used as a substitute for mineral oil. The results of this work effectively showed that the fractal dimension of the discharges is linked to the relative permittivity of the insulating materials and their thickness. This work also shows that the results of the analysis of the propagation of discharges in the vegetable oils investigated are close to 97%. Another of these papers focuses on the impact of ageing of the solid/liquid insulation system on the propagation of creeping discharges^13^. The liquids investigated include palm kernel oil methyl esters and mineral oil. The technique used here involves quantifying the rate of ionisation produced in the various liquids as a function of their ageing time. On the other hand, the impact of the ageing of the system on the maximum extension of discharges and the fractal dimension was evaluated. The results of this work show that the ageing of liquids leads to a considerable increase in the level of ionisation at the solid/liquid interface and can lead to total destruction of the cellulose beyond 600 h. The results show that the impact of ageing on the ionisation rate is greater in mineral oil. It is also shown that the fractal dimension of the discharges produced is linked to the ageing time of the liquids.

The work presented above has provided a considerable database for understanding and preventing the possible risks of destruction of power transformers due to the propagation of creeping discharges. However, the obvious observation that emerges from this work is that it is carried out at room temperature. In contrast, in a power transformer, the temperature variation in the insulation system depends on both the current draw by the actual load and the weather conditions during operation of the transformer. In the case of Class A transformers, for example, the maximum permissible temperature is 105 °C^14,15^. According to the IEEE C57.12.01-2015 standard, the maximum temperature of immersed transformers can be as high as 55 °C during full load operation. It should also be noted that, under extreme environmental conditions and in cases of overloading, the temperature of the upper part of the oil can exceed 90 °C. The aim of this research work is therefore to contribute to the existing database on the propagation of creeping discharges in vegetable and mineral oils when these are subjected to significant temperature stresses. In contrast to the previous work, this project also provides quantitative data on the rate of ionisation, the charge and the maximum extension of the branches that can be induced by a creeping discharge as a function of the type of liquid and the level of stress (temperature and voltage).

## Experimental details

### Preparation of impregnated pressboard samples

In this study, thermally enhanced Kraft (TUK) cellulose paper was used as a solid insulator. In accordance with IEC60641-2, paper samples were previously dried in a forced convection oven heated to 105 °C for 48 h. Then at low pressure at 85 °C for 48 h. The insulating liquids used in this study are: mineral oil (MO), palm kernel oil methyl ester (PKOME) and castor oil methyl ester (COME). These monoesters were obtained by a degumming and transesterification process fully detailed in previous open access publications^16,17^. After chemical treatment, the liquids were also dried for 48 h at 85 °C before introducing the paper samples. The assembly was returned to the oven and heated to 85 °C for 48 h before use. Tables 1 and 2 show some of the physicochemical and thermal characteristics of these liquids as presented in previous publications. Table 3 shows the characteristics of TUK paper.

**Table 1:** Physicochemical characteristics of the insulating liquids used^18^.

Characteristic	Unit	Standard	MO	COME	PKOME
Density at 20 °C	–	ASTM D1217	0.857	0.924	0.890
Kinematic viscosity at 40 °C	cSt	ASTM D445	9.65	15.06	4.87
Kinematic viscosity at 100 °C	cSt	ASTM D445	1.20	3.14	1.90
Total Acid Nomber	mg KOH/g	ASTM D974	0.05	0.04	0.05
Flash point	°C	ASTM D92	146	183	167
Fire point	°C	ASTM D92	175	218	182
Breakdown voltage	kV	ICE 60156	68.66	74.77	87.68

**Table 2:** Thermal characteristics of the insulating liquids used^19^.

Temperature (°C)$\to$		25	40	60	80	100
Thermal conductivity (W.m^−1^ °C^−1^)$\downarrow$						
PKOME	ASTM D7876	0.180	0.175	0.166	0.158	0.150
COME		0.173	0.166	0.157	0.151	0.144
MO		0.133	0.130	0.128	0.126	0.120
Specific heat (J kg^−1^ °C^−1^)$\downarrow$						
PKOME	Adiabatic calorimetry method	1500.1	1850	2140	2335	2541
COME		1523.2	1883	2182	2373	2565
MO		1820.0	1930	2000	2150	2195

**Table 3:** Characteristics of TUK paper^20^.

Property	Standard (ASTM)	TUK
Thickness (mm)	D645	0.08
Weight (g/cm^3^)	D646	0.89
Dielectric constant	D150	4.7
Dielectric dissipation Factor	D150	0.25%
AC BDV (kV/mm)	D149	10
Tensile strength, MD (N/cm)	D128	65

### Experimental set-up

**Figure 1:**
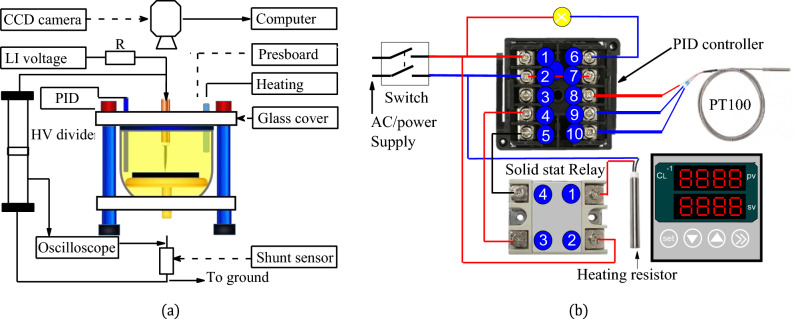
(**a**) Experimental set-up; (**b**) Connection of the temperature controller.

The experiment is carried out at a negative lightning impulse voltage provided by a Marx generator (200 kV–1.25 kJ, 1.2/50 μs). The impulse voltage was measured using a capacitive voltage divider and an oscilloscope. The volume of oil used for each phase of the experiment is 3 L. The test cell is a porcelain vessel with a capacity of 5 L and a wall thickness of 10 mm. The electrode system is of the point and plane type. The earth electrode, which forms the flat part, is made of brass with a diameter of 250 mm, a thickness of 30 mm and a radius of curvature of the periphery of 5 mm. The needle electrode, which is connected to the voltage wire, is made of tungsten with a tip radius of 50 μm. To illustrate the case of a triple junction in a power transformer, the tip electrode is placed in direct contact with the solid insulator. The discharges observed are therefore those occurring on the surface of the insulating paper. The oil is heated by a system consisting of a proportional integral derivative (PID: soobufwb40tk7rh-11) controller and a 1000 W heating resistor. The insulating paper used is cut and stacked to form a square pressboard 10 mm thick and 160 mm square. The image acquisition system for the discharges consists of a Panasonic GP-KR22 color CCD camera and a computer. To keep measurement errors to a minimum, the camera exposure time must be less than the lightning impulse shock wave. The data is processed using Matlab and ORIGIN software. Figure 1a,b show a full description of the experimental set-up and the temperature controller connection. It should be noted that, before any experiment, the temperature gradient was measured. This ensures that the electronic temperature measurement device is protected against strong eclectic fields. Indeed, when lightning impulses stress are applied, the PID detector is shut down for a few seconds on the basis of the temperature gradient.

### Assessment of areas of high ionization

**Figure 2:**
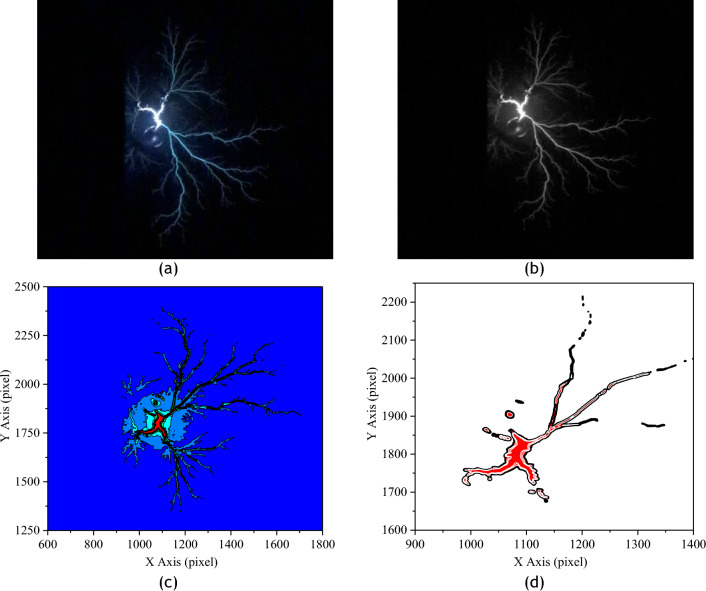
Extraction of high ionisation zones. (**a**) original image, (**b**) grey scale image (**c**) cartography, (**d**) segmentation.

The propagation of creeping discharges at the solid/liquid interface is generally associated with a luminescent phenomenon which intensity and surface distribution depend on the intensity of the electric field, the type of insulating liquid and the characteristics of the pressboard. This phenomenon marks the rate of ionisation in the insulation and can therefore be used as a characteristic parameter to describe the impact of a discharge on an insulating surface. Indeed, experience has shown that a very large impact field has the effect of irreversibly destroying the insulating surface^12^. The aim of this section is therefore to quantitatively assess the impact of temperature on the ionisation rate in the vegetable oils investigated, compared with mineral oil. This data is obtained in four steps. Firstly, the original image is transformed to obtain its greyscale equivalent. From this, the intensity of the pixels in the image is mapped using OriginPro software. Now that the areas of high intensity are visible, these are segmented and the number of pixels in them is counted. The final results are the average of ten images obtained under the same experimental conditions with a relaxation time of 5 min. They are compared for each insulating liquid and as a function of temperature stress and for two voltage levels. Figure 2a–d illustrate the steps involved in extracting ionising pixels of a discharge.

### Maximum length

**Figure 3:**
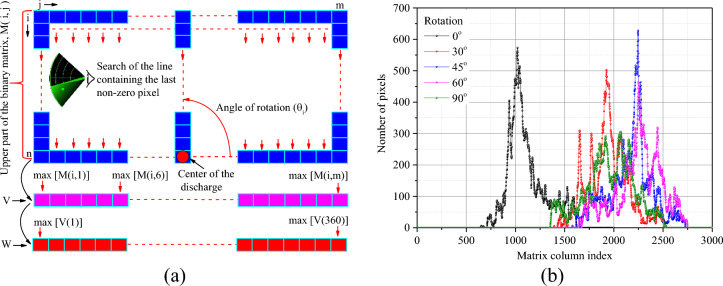
Algorithm for calculating the extension of discharges.

The algorithm for calculating the maximum extension of discharges is based on binarisation, skeletonisation, geometric operations and pixel counting techniques. The first step consists of converting the original image into its binary equivalent. The second step consists of skeletonising the image so as to retain only the average line of the image, made up of a single consecutive alignment of pixels. We define a matrix *M* representing the upper part of the skeleton image taken from the center of the discharge. The algorithm reads all the columns of this matrix, looking for the column index of the last non-zero pixel. This index data is progressively stored in a vector *V*. The algorithm then searches for the maximum of the vector V and stores the data in a new vector named *W*. The program then rotates the image with an angular step of one degree and repeats the previous process until it covers a complete 360° rotation. Note that each rotation involves resizing the image in order to retain the proportions of the original image and avoid any measurement errors. Once this stage is complete, the maximum of the vector *W* represents the pixel size of the longest branch of the discharge. This data can be converted into mm. To do this, it is multiplied by a calibration coefficient *n* obtained from the position of the camera and a measurement previously taken on an image of known dimensions. The final maximum length is the average of the data from ten images obtained under the same experimental conditions. Figure 3a shows an illustration of this algorithm and Fig. 3b shows an example of the content of the vector *V* as a function of the angle of rotation of the image.

### Total charge generated by the discharge

**Figure 4:**
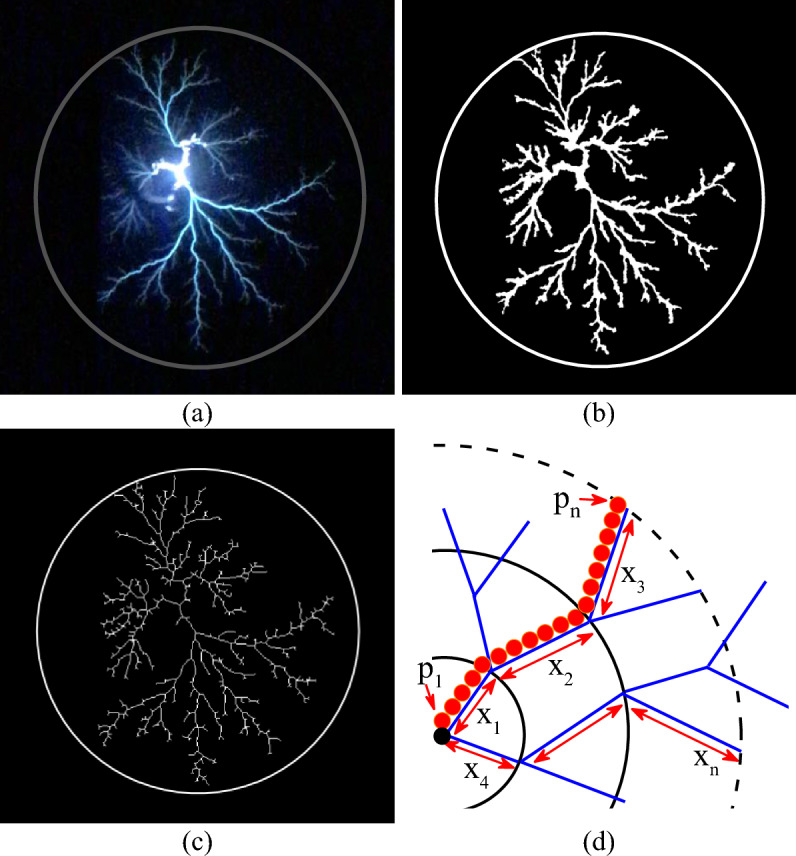
Illustration of the method for calculating the total length of discharges; (**a**) original image, (**b**) binary image, (**c**) Skeleton, (**d**) illustration of the skeleton structure^12^.

To calculate the charge associated with creeping discharges under a combined voltage and temperature constraint, the algorithm is based on the model initially introduced by Atten and Saker^21^ and revisited by Kebbabi et al.^10^. In their model, the first authors consider the discharge as a single streamer propagating at the liquid/solid interface. They assume that the channel of the constant-radius streamer is at a constant potential U and express the charge according to Eq. (1). In the vertical tip experiments and under negative voltage, the branches are more numerous with a radial filamentary shape. This justifies the revised model of Kebbabi et al.^10^ given in Eq. (2), which is similar to a superposition of charges generated by each channel individually. The particularity of this work rests on the technique used to obtain the total length of the branches used to calculate the charge, as this is not clearly explained in the literature. As in the case of calculating the maximum extension of discharges, this technique involves the binarisation and skeletonisation of the original image. As the image skeleton is made up of a continuous association of single pixels forming the average line of the binary image, the total length is obtained by summing the non-zero pixels in it. This is converted into millimeters by multiplying it by the coefficient n explained above. Figure 4a–c show an example of the image skeletonisation process. Figure 4d is a more detailed illustration of the image obtained after a process of skeletonization of the binary image. Each circle in this image represents the continuous association of pixels ∑P_i_ contained in the line ∑x_i_ of the discharge. By combining this technique with the model of Kebbabi, we finally obtain the total charge from Eq. (3).

1 $$Q_{s} = g\frac{{2\pi \varepsilon_{o} \varepsilon_{r} }}{{{\text{ln}}\left( {\frac{2e}{{r_{c} }}} \right)}}Ux_{m}$$

2 $$Q_{T} = \frac{{2\pi \varepsilon_{o} \varepsilon_{r} }}{{{\text{ln}}\left( {\frac{2e}{{r_{c} }}} \right)}}UL_{T}$$

3 $$Q_{T} = \frac{{2\pi \varepsilon_{o} \varepsilon_{r} }}{{\ln \left( {\frac{2e}{{r_{c} }}} \right)}}U\left( {n\sum P_{i} } \right)$$

where e is the thickness of the solid insulator; x_m_ is the length of the streamer; g is a constant reflecting the influence of the surrounding of the channel (g ≤ 1). L_T_ is the total length of all branches. In this study, the average radius of a branch (r_c_) was determined manually directly in the Matlab software for a series of 20 images. This was 72.23 μm. *n* is the unit length conversion factor. Pi represents non-zero pixels.

### Ethical approval

The authors of this papers fully applied the rules of ethics during the experiments and the writing of the paper.

## Experimental results

### Effect of temperature on ionization

**Figure 5:**
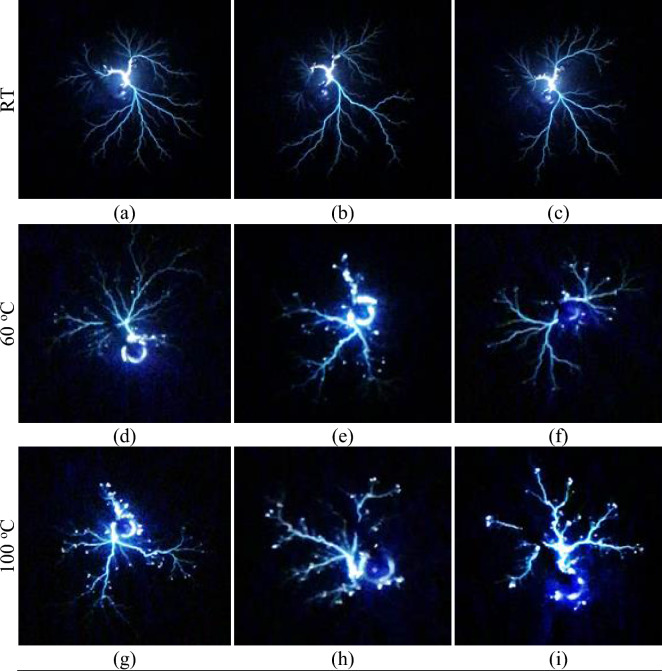
Propagation of discharges in each liquid at 90 kV as a function of temperature.

**Figure 6:**
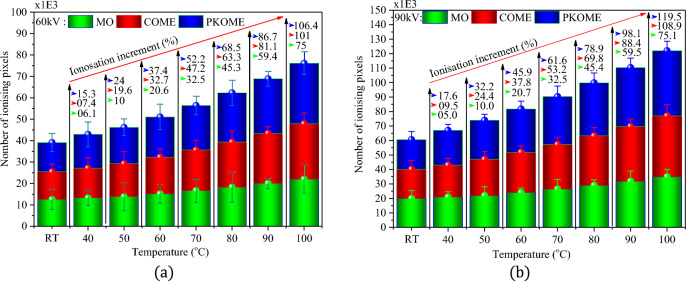
Ionisation level in each liquid at 60 kV (**a**) and 90 kV (**b**).

**Figure 7:**
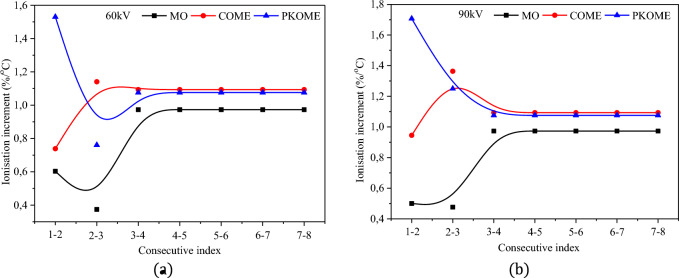
Ionisation increment per degree Celsius at 60 kV (**a**) and 90 kV (**b**).

Visual observation at 1/4 scale of the images at room temperature (RT) of creeping discharges obtained by optical detection show a disproportionate distribution of the ionisation phenomenon before the application of temperature stress (Fig. 5a–c). It can be seen that the phenomenon is more pronounced in the vicinity of the source of the discharge. This result shows that the point of impact of the discharge represents the region of greatest risk for transverse rupture of the dielectric insulator due to the high intensity of the electric field at this point. This interpretation was actually observed during the experiments, which led to the use of a large number of samples in order to guarantee the reliability of the final result. A similar result on ionisation at the point of impact is reported by Dang et al.^5^. As soon as the temperature is applied, the photographs of the discharges obtained are completely different. Visual observation at 1/5 scale (Fig. 5d–i) shows an extension of the ionisation phenomenon from the center of the discharge towards the branches. This can be seen in the appearance of granules on the branches. This phenomenon becomes progressively more pronounced as the temperature rises, a priori independently of the type of insulating liquid used. However, quantitative studies of this phenomenon in esters and mineral oil have shown that it is not completely independent of the physicochemical characteristics of the liquids. Figure 6a,b show an example of the average ionisation rate obtained in each of the liquids as a function of temperature and for two voltage levels. These results show that the temperature-related increase in ionisation level is greater in esters than in mineral oil. The results at 40 °C and 60 kV are 6.1, 7.4 and 15.3% respectively for MO, COME and PKOME. Under the same experimental conditions at 90 kV, these results are 5, 9.5 and 17.6% respectively in MO, COME and PKOME. An analysis of the ionisation increment per degree of temperature was also carried out in each of the liquids. Unlike the previous increment results, where the data are compared with those obtained at room temperature, these are obtained between consecutive and increasing temperature values. The results of this analysis presented in Fig. 7a,b show that, for temperature levels below 50 °C, the increment data changes as in a transient regime. However, above 50 °C the data become constant. This regime, which closely resembles a steady state, gives constant increments of 0.973%/°C, 1.093%/°C and 1.076%/°C in MO, COME and PKOME respectively. This result shows that a combined voltage and temperature constraint creates a greater negative impact in terms of ionisation.

### Maximum extension of discharges

**Figure 8:**
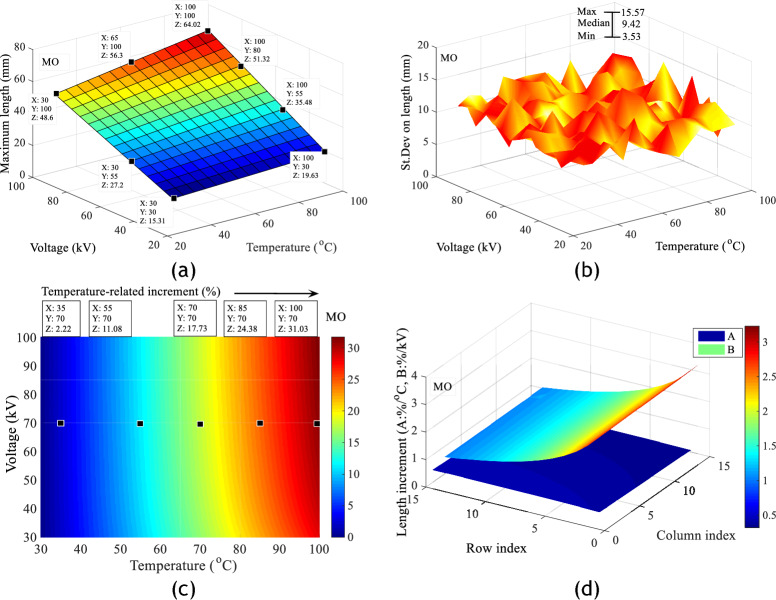
Impact of combined voltage and temperature stress on discharge extension in MO; (**a**) Length increment, (**b**) Standard deviation on length, (**c**) Length increments as a function of temperature at constant voltage, (**d**) Branch propagation gradient under combined stresses.

**Figure 9:**
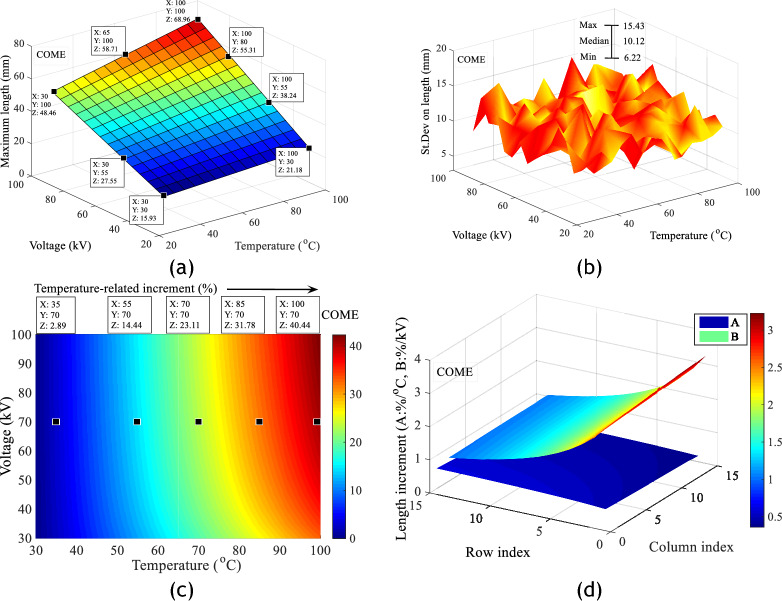
Impact of combined voltage and temperature stress on discharge propagation in COME; (**a**) Length increment, (**b**) Standard deviation on length, (**c**) Length increments as a function of temperature at constant voltage, (**d**) Branch propagation gradient under combined stresses.

**Figure 10:**
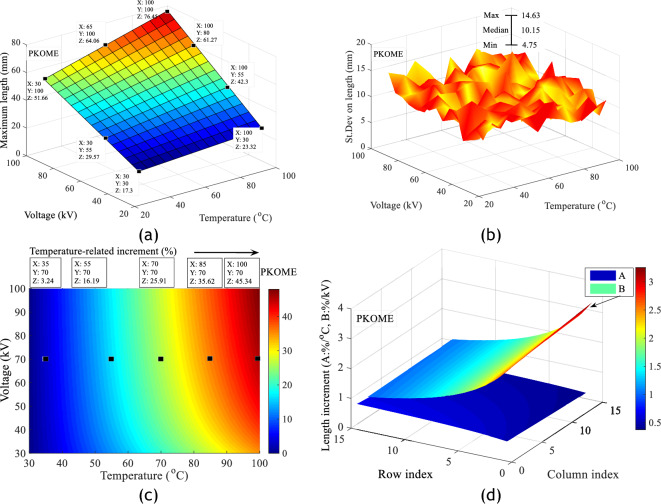
Impact of combined voltage and temperature stress on discharge propagation in PKOME; (**a**) Length increment, (**b**) Standard deviation on length, (**c**) Length increments as a function of temperature at constant voltage, (**d**) Branch propagation gradient under combined stresses.

Analysis of the data from the images of the discharges obtained in the different liquids shows that these evolve almost linearly with the applied voltage and temperature. However, for a given voltage level, the extension of the discharges is greater in PKOME and COME than in MO. This result is similar to those demonstrated by Beroual et al in a similar study carried out at room temperature. In their studies, they showed that there is a linear relationship between the length of the discharges and the applied voltage. They also show that insulating liquids with a lower dielectric constant have a better ability to stop the propagation of creeping discharges. This hypothesis seems to be borne out by the permittivities of the liquids investigated. These are 2.2, 2.37 and 3.2 respectively for MO, COME and PKOME. On the other hand, the results obtained with combined voltage and temperature stresses have a negative impact on the propagation of discharges in all the liquids concerned. However, it can be seen that the vegetable oils investigated are the most affected by changes in temperature. Figure 8a,b show the fit data for the maximum extension of discharges in MO with an average fit coefficient of 0.989, and the standard deviations obtained for ten images at each measurement point under the same experimental conditions. Figure 8c,d show respectively, the increments of the maximum extension of the discharges as a function of temperature when the voltage is kept constant and the gradient in %/°C in MO. Figure 9a–d show the same results in COME in exactly the same order. Figure 10a–d show the same results in PKOME in exactly the same order. These increments are calculated with reference to the values obtained at room temperature. The average maximum extension data is initially stored in a matrix named L, where the columns represent temperatures and the rows represent voltages. The matrix of increments is then calculated according to Eq. (4) where *i* and *j* are natural numbers. The variation between 1 and 15 comes from the temperature going from its room value to 100 °C with a step of 5 °C which corresponds to 15 values. This matrix is obtained by comparing consecutive values at increasing temperatures and constant voltage, as shown in Eq. (5).

4 $$Increment_{i,j} \left( \% \right) = \left\{ \begin{gathered} 1 \le i \le 15 \hfill \\ 2 \le j \le 15 \hfill \\ 100 \times \frac{{\left( {L\left( {i,j} \right) - L\left( {i,1} \right)} \right)}}{{L\left( {i,1} \right)}} \hfill \\ \end{gathered} \right.$$

5 $$Increment_{i,j} \left( \% \right) = \left\{ {\begin{array}{*{20}c} {1 \le j \le 15} \\ {2 \le i \le 15} \\ {100 \times \frac{{\left( {L\left( {i,j} \right) - L\left( {i - 1,j} \right)} \right)}}{{5 \times L\left( {i - 1,j} \right)}}} \\ \end{array} } \right.$$

These results show, from a few measurement points arbitrarily placed on Figs. 8c, 9c and 10c, that temperature has more negative effects on COME and PKOME than on MO. We can see that at 100 °C we have increments of 31.03, 40.44 and 45.34% respectively in MO, COME and PKOME. It can also be seen that although the increment per degree Celsius does not show a linear trend, it increases as the temperature rises.

**Figure 11:**
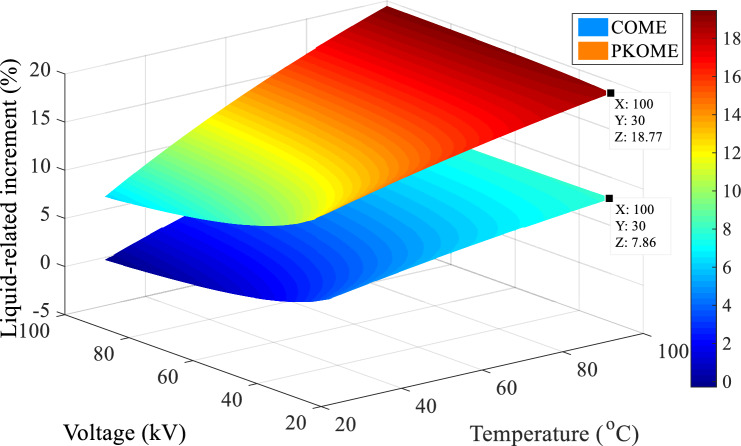
Increment of discharge extension in COME and PKOME compared to MO.

The last matrix is that which compares the maximum extension data of the same coordinates (Voltage, Temperature) obtained in the mineral oil and the esters, the reference values being those of the mineral oil. The algorithm for calculating the increment matrix in this case is given by Eq. (6). The results of these operations presented in Fig. 11 show that, before the temperature constraint, the maximum difference in length between MO and COME is 4.1%, and 13% between MO and PKOME. This result is close to those found in previous work at room temperature^12^. However, these data also show that an increase in temperature stress is disadvantageous for esters. We observed a difference of up to 7.86% between MO and COME and 18.77% between MO and PKOME for a temperature of 100 °C.

6 $$Increment_{i,j} \left( \% \right) = \left\{ {\begin{array}{*{20}c} {1 \le i \le 15} \\ {1 \le j \le 15} \\ {100 \times \frac{{\left( {L_{ester} \left( {i,j} \right) - L_{MO} (i,j} \right))}}{{L_{MO} \left( {i,j} \right)}}} \\ \end{array} } \right.$$

### Charge generated

**Figure 12:**
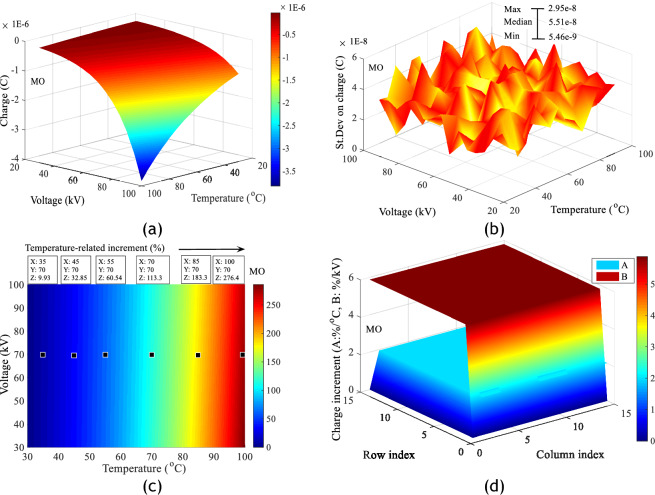
Impact of combined voltage and temperature stress on charge increment in MO, (**a**) Charge increment, (**b**) Standard deviation on charge, (**c**) Charge increments as a function of temperature at constant voltage, (**d**) Charge gradient under combined stresses.

**Figure 13:**
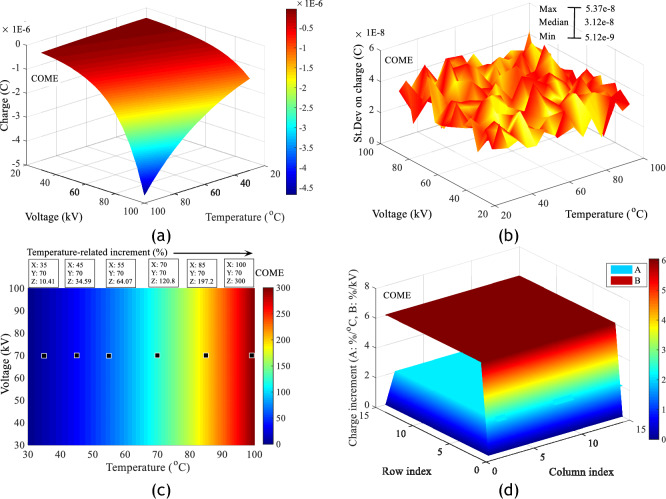
Impact of combined voltage and temperature stress on charge increment in COME, (**a**) Charge increment, (**b**) Standard deviation on charge, (**c**) Charge increments as a function of temperature at constant voltage, (**d**) Charge gradient under combined stresses.

**Figure 14:**
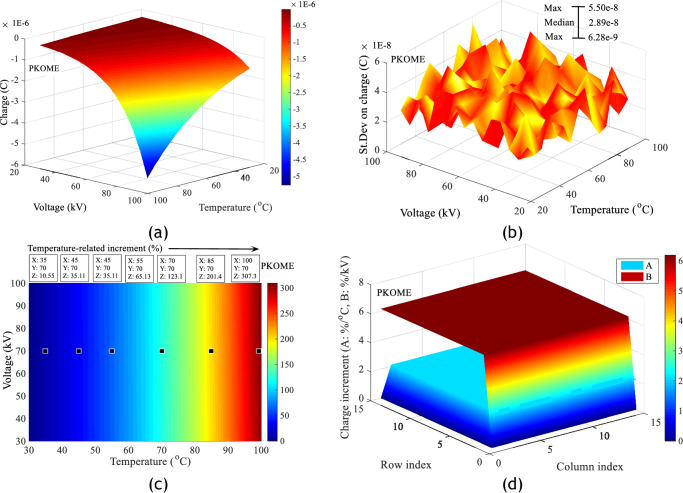
Impact of combined voltage and temperature stress on charge increment in PKOME, (**a**) Charge increment, (**b**) Standard deviation on charge, (**c**) Charge increments as a function of temperature at constant voltage, (**d**) Charge gradient under combined stresses.

A study of the charge produced in each sample of insulating liquid shows that temperature increases the risk of deterioration of the insulating surface. This phenomenon is all the more important when the combined stress (Temperature, Voltage) is high. Figure 12a–d shows respectively the charge adjustment matrices obtained in each of the insulating liquids under combined stresses with average adjustment coefficients of 0.989, the standard deviations of the measurements, the increment values of the charges and charge gradient in %/°C in MO. Similar results in COME and PKOME with respective adjustment coefficients of 0.995 and 0.992 are presented in the same order as above in Figs. 13a–d and 14a–d. These values are calculated in relation to those obtained at room temperature. These are calculated between consecutive values at increasing temperature. All the matrices presented in this section are calculated according to Eqs. (7) and (8). As in the case of the maximum extension of the discharges, these results show that the esters investigated are more affected by the temperature constraint than the mineral oil. For a few arbitrarily chosen measurement points, we observe increments at 100 °C of 276.4, 300 and 307.3% respectively in MO, COME and PKOME. Similarly, the gradient (%/kV and %/kV) is almost constant in all liquids. The incremental values are (5.839%/°C, 1.977%/kV), (6.047%/°C, 2.082%/kV) and (6.177%/°C, 2.113%/kV) respectively in MO, COME and PKOME.

7 $$Increment_{i,j} \left( \% \right) = \left\{ {\begin{array}{*{20}c} {1 \le i \le 15} \\ {2 \le j \le 15} \\ {100 \times \frac{{\left( {C\left( {i,j} \right) - C\left( {i,1} \right)} \right)}}{{C\left( {i,1} \right)}}} \\ \end{array} } \right.$$

8 $$Increment_{i,j} \left( \% \right) = \left\{ {\begin{array}{*{20}c} {1 \le j \le 15} \\ {2 \le i \le 15} \\ {100 \times \frac{{\left( {C\left( {i,j} \right) - C\left( {i - 1,j} \right)} \right)}}{{5 \times C\left( {i - 1,j} \right)}}} \\ \end{array} } \right.$$

**Figure 15:**
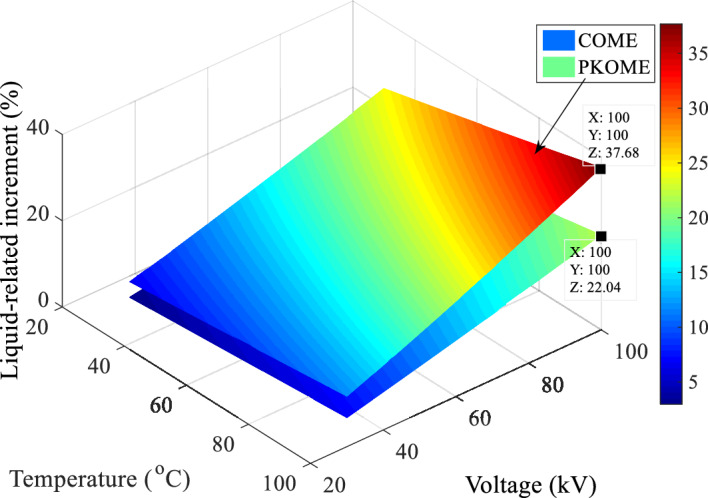
Charge increment in COME and PKOME compared to MO.

The last comparison is between the charges generated by the creeping discharges. This is done between the data with the same coordinates (Voltage, Temperature) obtained in the mineral oil and the esters, the reference values being those of the mineral oil. The algorithm for calculating the increment matrix in this case is the same as that used in the case of maximum extension and given by Eq. (9). The results of these operations, presented in Fig. 15, show that before temperature stress, the maximum difference in charge between MO and COME is 12.77, and 24.27% between MO and PKOME. At a temperature of 100 °C, the difference between MO and COME was 22.24% and between MO and PKOME 37.68%.

9 $$Increment_{i,j} \left( \% \right) = \left\{ {\begin{array}{*{20}c} {1 \le i \le 15} \\ {1 \le j \le 15} \\ {100 \times \frac{{\left( {L_{ester} \left( {i,j} \right) - L_{MO} (i,j} \right))}}{{L_{MO} \left( {i,j} \right)}}} \\ \end{array} } \right.$$

## Discussions

The various results obtained in this work can be explained by the two main phenomena involved in this process. These include the phenomenon of charge transport linked to molecular dissociation under thermal stress and the phenomenon of ionisation due to the charge injection process and amplified by the excess charge resulting from molecular dissociation. In dielectric liquids, charge transport under temperature stress is closely linked to conductivity, which in turn depends mainly on impurity ions with low mobility at room temperature^22^. The parameters that influence electrical conductivity include temperature and ionic activation energy, which in turn depends on the external electric field. The relationships between the electrical conductivity of a liquid and the electric field and ion density are given in Eqs. (10–13)^23^.

10 $$\gamma = \frac{j}{E} = n_{0} q\mu$$

11 $$n_{0} = A_{0} {\text{exp}}\left( {\frac{{\Delta u_{a} - u_{o} }}{2kT}} \right)$$

12 $$\mu = \left( {q\delta^{2} v/6{\text{kT}}} \right){\text{exp}}\left( { - \frac{{u_{0} }}{kT}} \right)$$

13 $$\Delta u_{a} = \sqrt {q^{3} E/\pi \varepsilon \varepsilon_{0} }$$

In which j represents the current density, E the external electric field strength, q the ion, μ represents the mobility of the ion, ${{\varvec{n}}}_{0}$ the density of the ion in the liquid dielectric. ${{\varvec{u}}}_{{\varvec{a}}}$ is the activation energy, ${\varvec{\varepsilon}}$ is the dielectric constant of the liquid dielectric, ${{\varvec{\varepsilon}}}_{0}$ represents the permittivity of vacuum, δ is the mean distance of the ion transition, ${{\varvec{u}}}_{0}$ is the potential barrier of the ion, v is the vibrational frequency of the ion.

It can be seen from the above relationships that the ionic density and mobility in the insulating liquid increases with increasing temperature. Volume resistivity can also be calculated according to Eqs. (14–16).

14 $$\rho = \left( {\frac{6kT}{{A_{0} q^{2} \delta^{2} v}}} \right){\text{exp}}\left( {\frac{{2u_{0} + u_{a} - \sqrt {q^{3} E/\pi \varepsilon \varepsilon_{0} } }}{2kT}} \right)$$

15 $${\text{Assuming}}\;{\varvec{A}} = \left( {\frac{{6{\varvec{kT}}}}{{{\varvec{A}}_{0} {\varvec{q}}^{2} {\varvec{\delta}}^{2} {\varvec{v}}}}} \right),\;{\mathbf{B}} = \left( {\frac{{2{\varvec{u}}_{0} + {\varvec{u}}_{{\varvec{a}}} }}{{2{\varvec{k}}}}} \right),\;{\text{and}}\;{\varvec{C}} = \left( {\frac{{\sqrt {{\varvec{q}}^{3} {\varvec{E}}/\user2{\pi \varepsilon \varepsilon }_{0} } }}{{2{\varvec{k}}}}} \right)$$

We obtain

16 $$\user2{ln\rho } = {\varvec{lnA}} + \frac{{\varvec{B}}}{{\varvec{T}}} - {\varvec{C}}\sqrt {\varvec{E}} /\user2{T,}\;\frac{{\partial \left( {\user2{ln\rho }} \right)}}{{\partial {\varvec{T}}}} = - \frac{{{\varvec{B}} - {\varvec{C}}\sqrt {\varvec{E}} }}{{{\varvec{T}}^{2} }} < 0\;{\text{and}}\;\frac{{\partial \left( {\user2{ln\rho }} \right)}}{{\partial {\varvec{E}}}} = - \frac{{0.5{\varvec{C}}}}{{{\varvec{T}}^{2} {\varvec{E}}^{ - 0.5} }} < 0$$

So, according to the last relationship, volume resistivity decreases with increasing electric field strength and temperature. In addition, it should be noted that at room temperature this value is 1000 times greater in mineral oils than in vegetable oils^24^. This contributes to a greater weakening of the activating energy in the vegetable oils.

Equation (13) involving the activation energy is proportional to 1/√ε under the assumption that the electrical stress is the same for each measurement point in the different liquids. This shows if we refer to the different permittivities of the liquids investigated in this study that, the ionic activation energy is higher in MO and followed by COME and PKOME respectively. The activation energy is a form of potential barrier that conditions the release and diffusion of charged ions in the liquid. This implies that even at room temperature, the ionic density is greater in PKOME and COME than in MO in that respective order. This becomes all the more true when the liquids are subjected to significant temperature increases. The order of magnitude of the electrical conductivity of vegetable oils can be 100 times that of mineral oil^25^, and these are even higher under the effect of temperature. This will have the effect of considerably increasing the density of free ions in each of these liquids. Fubao et al.^23^ have also shown that under high temperature conditions, some of the charge contained in the paper diffuses into the oil, leading to an increase in the free charge density and an increase in the electric field intensity. However, it is well known that the propagation of creeping discharges in insulating liquids with a point and plane geometry involves a charge injection process^26^. In order to highlight the relationship between the significant densification of the quantity of ions under thermal stress and the propagation of discharges, it is necessary to detail the processes involved. The process of injecting charge into the liquid starts at the needle electrode. To do this, the electrons in the needle's conduction band must be able to reach its surface and have enough energy to overcome the needle's work function before propagating into the liquid^27^. This refers to the energy required to pull an electron from the surface of a metal. When this condition is met, the electrons are released by the needle electrode and begin to propagate into the liquid. Under room temperature conditions, the electrons must be injected with a sufficiently strong electric field to create collisions and increase the vibrational energy of the molecules, which will then cause an avalanche effect and local expansion^27^. This explains why the branch lengths, charge and ionisation rate are lower at low temperatures in this work. A progressive increase in temperature creates a progressive increase in the vibratory energy of the molecules and we have an "ionic pollution" of the liquid as presented at the beginning of the discussion. This implies, on the one hand, that the electric field required to create collisions and local expansion by avalanche necessarily becomes lower. On the other hand, we can see that the number of collisions will be greater for liquids with greater "ion pollution". This justifies, on the one hand, the increase in ionisation and other parameters characteristic of creeping discharges when the combined stress (temperature, voltage) is seen to increase in the liquids. And on the other hand, the differences observed between the results in each liquid.

Despite the significant impact of temperature on the characteristic parameters of the discharges contained in COME and PKOME, these seem rather promising for possible use in power transformers as a substitute for mineral oil. Indeed, based on the characteristics of the vegetable oils investigated in this way at room temperature, the impact on COME and PKOME remains relatively low. For example, we have the work of Beroual et al.^28^ on the comparative study of these phenomena in mineral oil and vegetable oils extracted from grape seeds (GS), sunflower (SF) and rapeseed (RS). At the end of this work, it was concluded that these investigated vegetable oils present maximum discharge extensions whose values are 60% and 40% higher than MO in positive and negative voltage respectively. The same finding was reported by Sitorus et al.^6^ in their comparison of Jatropha curcas methyl ester (JMEO) with MO. His work shows that discharges in vegetable oil have extensions whose values are 60% higher than those in MO. A number of results in this direction can be found in the literature^29,30^. Even at high voltage and temperature, the results obtained with COME and PKOME show increments that are much lower than those found in the literature at room temperature.

## Conclusions

In a power transformer, failures linked to leakage currents occur most of the time when the transformer is in full operation. This means that temperature and voltage are constrained in real time. However, the vast majority of work carried out in the search for plant-based liquids as an alternative resource to mineral oils is conducted at room temperature. The aim of this work was therefore to study the impact of a combined voltage and temperature constraint on the characteristic parameters of creeping discharges propagating along cellulose insulation immersed in vegetable oils (palm kernel and castor oil methyl esters), compared with mineral oil. The analysis was carried out by evaluating the rate of ionisation, the maximum extension of the discharges and the charge produced in each liquid under different levels of combined stress. The results of the study show that:

- At relatively low temperatures, castor oil methyl ester (COME) has characteristics close to those of mineral oil (MO), unlike palm kernel oil methyl ester (PKOME).
- For temperatures greater than or equal to 50 °C, a constant ionisation gradient of 0.973%/°C, 1.093%/°C and 1.076%/°C is observed in MO, COME and PKOME respectively.
- The analysis of the impact of the combined stress of temperature and voltage on the charge produced in the different liquids gives the following results: (5.839%/°C, 1.977%/kV), (6.047%/°C, 2.082%/kV) and (6.177%/°C, 2.113%/kV) respectively for MO, COME and PKOME
- The branch lengths of the discharges produced in liquids increase as the temperature rises.
- Liquids with a high dielectric constant and electrical conductivity favor the propagation of discharges at high temperatures.
- Despite the significant impact of temperature on the characteristic parameters of the discharges contained in COME and PKOME, these seem rather promising for possible use in power transformers as a substitute for mineral oil.

## IUCN policy statement on research involving species at risk of extinction

The seeds used to make the esters were grown on site, in the Littoral region of Cameroon, using all the methods required by regulations.

## Data Availability

All data and materials are available without restrictions, the corresponding author JLJ is the person to contact in case of need.
